# Supplementary material for: Bio-inspired poly-DL-serine materials resist the foreign-body response
Source: Nat Commun. 2021 Sep 7;12:5327. doi: 10.1038/s41467-021-25581-9 (PMC8423817; doi:10.1038/s41467-021-25581-9)
Supplement: Supplementary file 1 — Supplementary Information [file 41467_2021_25581_MOESM1_ESM.pdf]

Supplementary Information

Bio-inspired poly-DL-serine materials resist the foreign-body response

Donghui Zhang[1], Qi Chen[2], Yufang Bi[2], Haodong Zhang[2], Minzhang Chen[2], Jianglin Wan[2], Chao Shi[2], Wenjing Zhang[2], Junyu Zhang[2], Zhongqian Qiao[2], Jin Li[3], Shengfu Chen[4], Runhui Liu[1, 2]*

[1]State Key Laboratory of Bioreactor Engineering, East China University of Science and Technology, Shanghai 200237, China

[2]Key Laboratory for Ultrafine Materials of Ministry of Education, Frontiers Science Center for Materiobiology and Dynamic Chemistry, Research Center for Biomedical Materials of Ministry of Education, School of Materials Science and Engineering, East China University of Science and Technology, Shanghai 200237, China

[3]Shanghai Key Laboratory of Orbital Diseases and Ocular Oncology, Department of Ophthalmology, Ninth People's Hospital, Shanghai Jiao Tong University School of Medicine, Shanghai 200011, China

[4]Key Laboratory of Biomass Chemical Engineering of Ministry of Education, College of Chemical and Biological Engineering, Zhejiang University, Hangzhou, Zhejiang 310027, China

Correspondence should be addressed to R.L. (rliu@ecust.edu.cn)

Supplementary Methods

Materials

O-tert-butyl-L-serine and O-tert-butyl-D-serine were obtained from Leyan®. Poly(ethyleneglycol) diacrylate (PEGDA), Mn = 2000 Da or 5000 Da were obtained from JenKem®. All other chemical reagents and solvents were purchased from Adamas-beta® and used without further purification. Information about antibody or kit are in the specific experimental sections. Water used in these experiments was obtained from a Millipore water purification system with a minimum resistivity of 18.2 MΩ cm. Ultraviolet lamp (NCSU033B, NICHIA) was purchased from Shenzhen Walker secret technology Co., Ltd.

Synthesis.

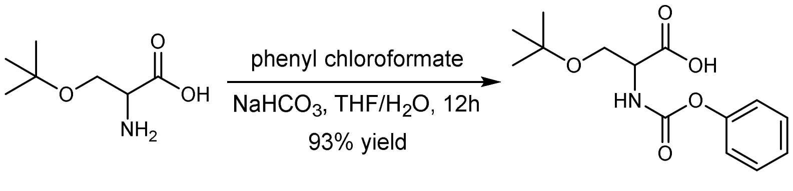

O-tert-butyl-L-serine (5 g, 31.0 mmol), O-tert-butyl-D-serine (5 g, 31.0 mmol) and sodium bicarbonate

(15.7 g, 186.1 mmol) were dissolved in a mixed solvent of THF (150 mL) and H_2O (150 mL). Then phenyl chloroformate (7.6 mL, 62.0 mmol) was slowly added to the above solution at 0 °C and the reaction mixture was stirred at room temperature for 12 h. The reaction mixture was concentrated under reduced pressure, re-dispersed in EtOAc/H_2O, and then adjusted with 1 N HCl to pH 2. After phase separation, the organic layer was washed with 10% citric acid and brine, dried over $MgSO_4$ and concentrated. The product was recrystallization from EtOAc/hexane to give a white crystalline solid (16.2 g, 93% yield). ^1H NMR (400 MHz, $CDCl_3$) δ 9.07 (s, 1H), 7.41-7.32 (m, 2H), 7.25-7.18 (m, 1H), 7.18-7.07 (m, 2H), 5.92 (d, J = 8.4 Hz, 1H), 4.64-4.48 (m, 1H), 3.96 (dd, J = 9.1, 3.1 Hz, 1H), 3.68 (dd, J = 9.1, 4.2 Hz, 1H), 1.21 (s, 9H). ESI-MS: $[M-H]^+$: 280.3.

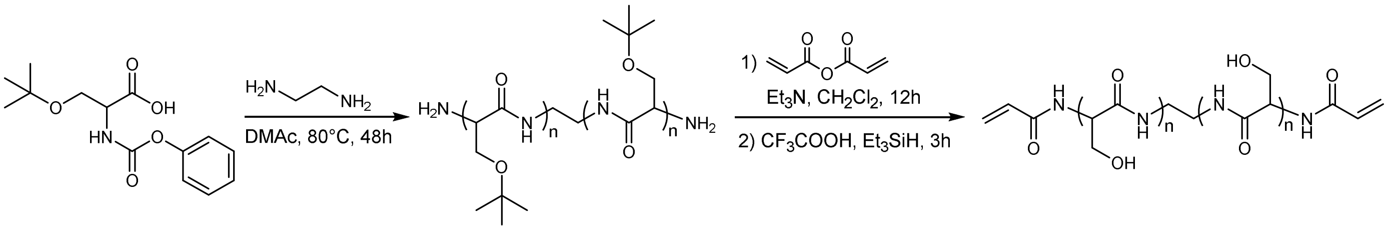

Polymers were synthesized using reported phosgene-free method.[1] 3-tert-butoxy-2(phenoxycarbonylamino)propionic acid (2.0 g, 7.11 mmol) was dissolved in dimethylacetamide (DMAc, 7 mL) in an dried flask. To the flask was added the ethylenediamine (22.6 µL, 0.34 mmol for short chain polymer; 8.6 µL, 0.13 mmol for long chain polymer). After stirred for 48 h at 80 °C, the mixture was poured into 300 mL methyl tert-butyl ether and the resulting polymer was precipitated out. The solid was isolated by filtration and washed with methyl tert-butyl ether thoroughly, followed by drying under vacuum. Samples of the protected diamine polyserine products (3 mg) were dissolved in a mixture of hexafluoroisopropanol/THF 1:3 (v/v, 1 mL) for Gel permeation chromatography (GPC) analysis on a Waters GPC instrument (with Breeze 2 software) equipped with a Waters 1515 isocratic HPLC pump and a refractive index detector (Waters 2414), using THF as the mobile phase at a

flow rate of 1 mL/min at 40 °C. Relative number-average molecular weight (Mn) and dispersity index (Đ) were calculated from a calibration curve using polystyrene (PS) as standards.

The amine terminated polymer was dissolved in 10 mL CH_2Cl_2 followed by adding acrylic anhydride (195 μL, 1.70 mmol) and Et_3N (283 μL, 2.03 mmol) to the reaction flask. The reaction mixture was stirred overnight and concentrated. Then petroleum ether (PE, 50 mL) was added to the reaction container to precipitate out the polymers as a solid. After two more cycles of the dissolution/precipitation operation using 50 mL of THF/PE (1:49, v/v), the collected polymer was dried under vacuum. Deprotection of the polymer was achieved by treating the white solid in 20 mL trifluoroacetic acid (TFA) supplemented with 1.2 mL triethylsilane (Et_3SiH) under gentle shaking for 3 hours at room temperature. The resulting solution was partially volatilized by N_2 and dispersed in 1 mL MeOH. The mixture was poured into 45 mL cold methyl tert-butyl ether to precipitate out the polymer as a solid that was isolated by centrifugation. The polymer was dried under a stream of N_2 and subjected to two more cycles of dissolution/precipitation in 50 mL of methanol/ether (1:49 mL, v/v) and further drying under vacuum to give a white solid. The solid was then dissolved in 6 mL water, filtered through 0.45 μm polyether sulfone membrane, dialyzed against 100-500 Da cellulose ester membrane, and lyophilized to afford polyserine diacrylamide (PSerDA) as a white fluffy powder (353 mg for short chain polymer, 52% yield; 458 mg for long chain polymer, 71% yield). The deprotected PSerDA products were characterized by ^1H NMR on an AVANCE III 400 spectrometer with TopSpin software at 400 MHz, using D_2O as the solvent.

Supplementary Figures

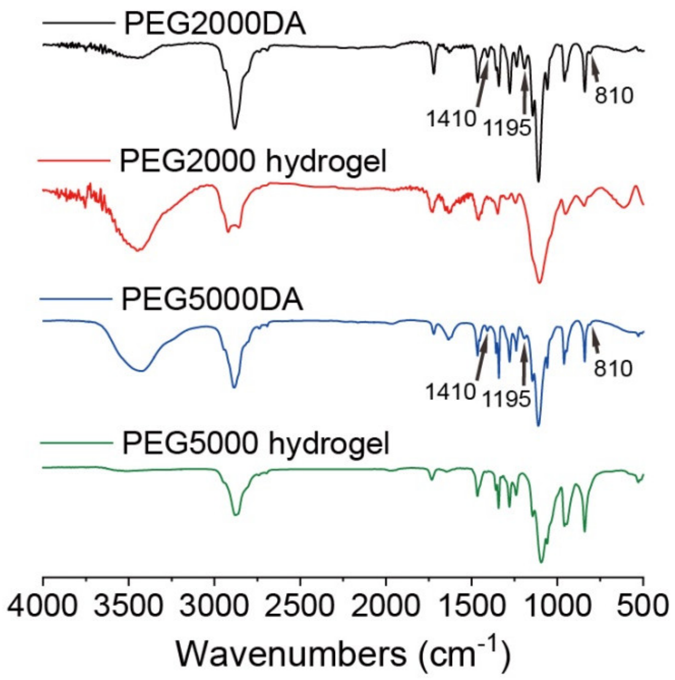

Supplementary Figure 1. FTIR spectra of PEGDA and PEG hydrogels.

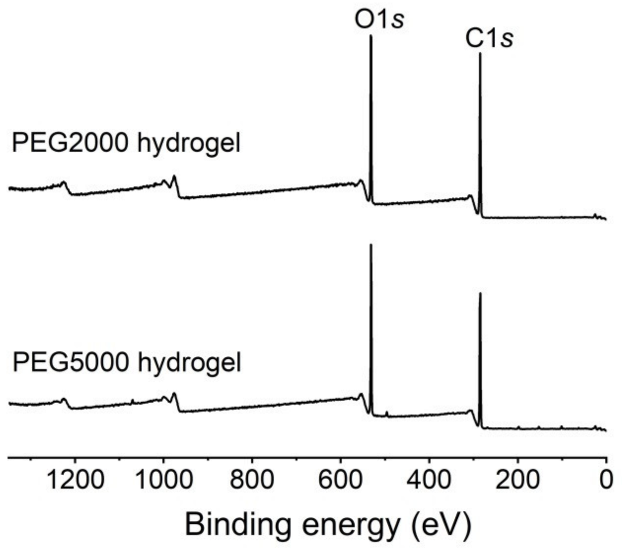

Supplementary Figure 2. XPS spectra of PEG hydrogels.

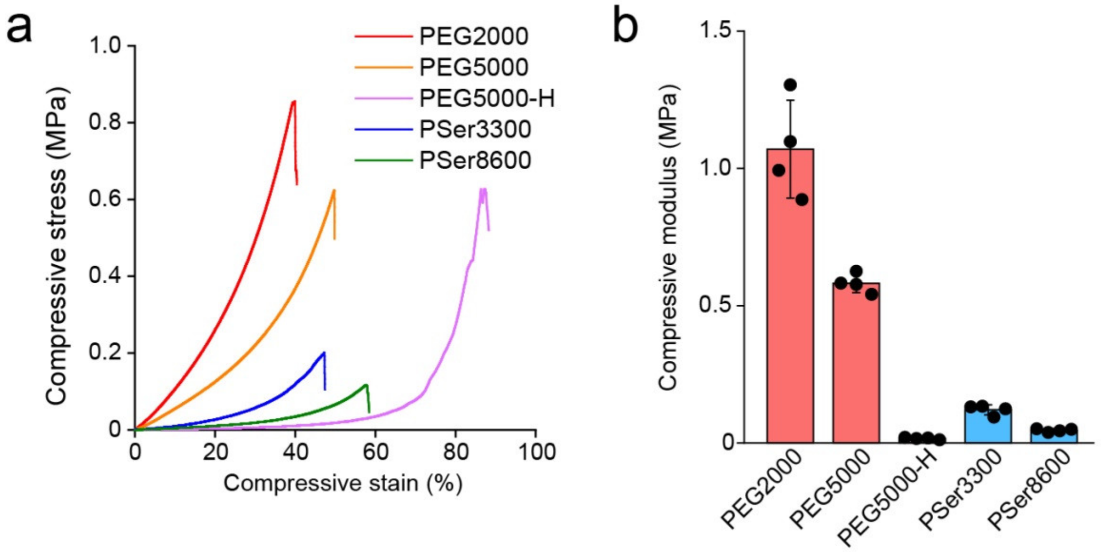

Supplementary Figure 3. a Compressive curves of hydrogels. **b** Compressive modulus of hydrogels. n = 4, mean values ± s.d.

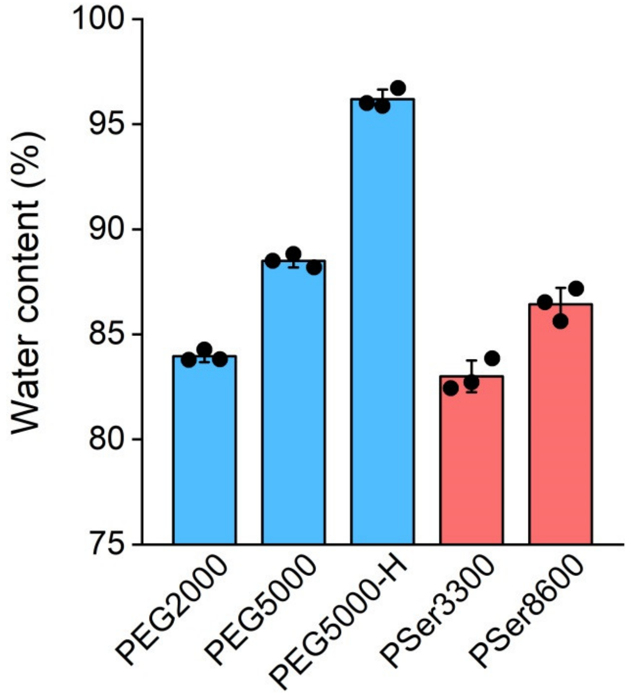

Supplementary Figure 4. Water content of hydrogels. n = 3, mean values ± s.d.

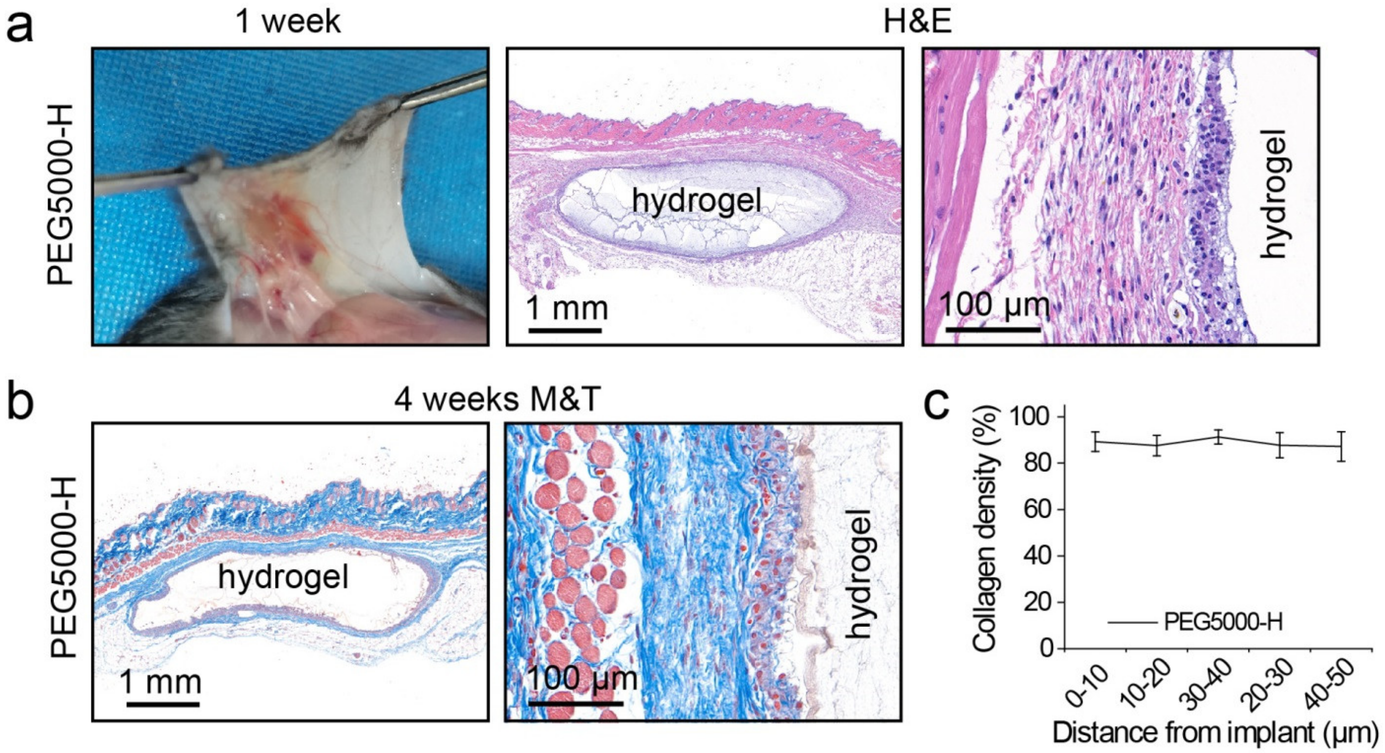

Supplementary Figure 5. a Explantation picture and H&E staining of PEG5000-H hydrogels after subcutaneously implanted in mice for 1 week. **b,c** M&T staining (**b**) and quantified collagen density (**c**) of PEG5000-H hydrogel-tissue interface after 4 weeks of implantation. n = 6, mean values ± s.d.

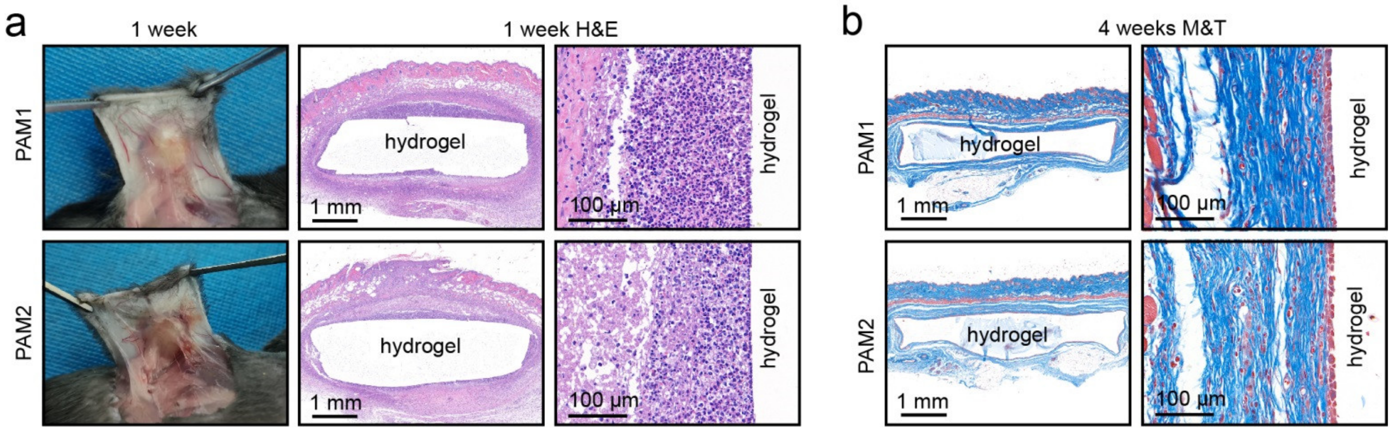

Supplementary Figure 6. a Explantation picture and H&E staining of polyacrylamide (PAM) hydrogels after subcutaneously implanted in mice for 1 week. **b** M&T staining of PAM hydrogels after 4 weeks implantation.

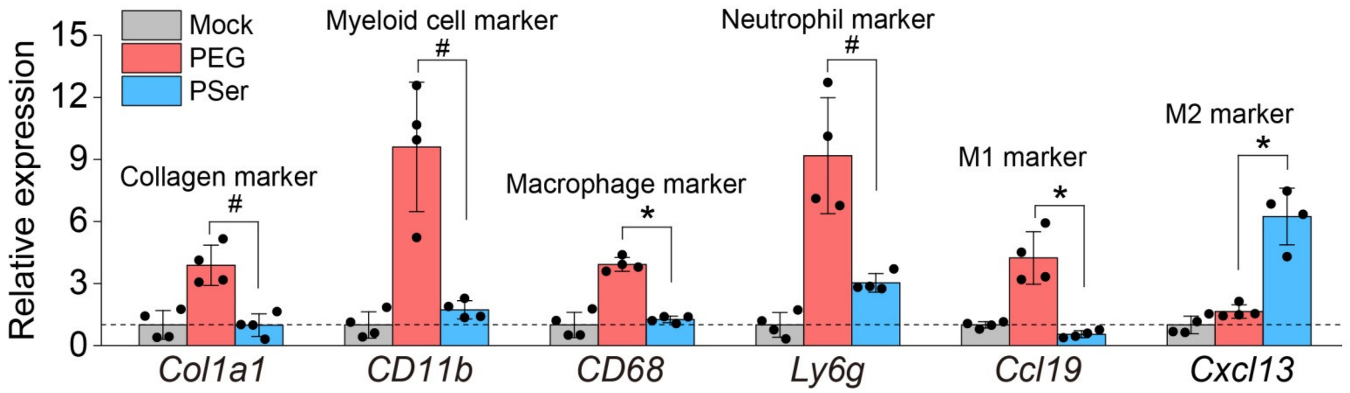

Supplementary Figure 7. qPCR assay. All materials were retrieved 2 weeks post implantation and assayed by gene expression to evaluate levels of different innate immune and fibrosis markers including *Col1a1* (collagen marker), *CD11b* (myeloid cell marker), *CD68* (macrophage marker), *Ly6g* (neutrophil marker), *Ccl19* (M1 macrophage marker) and *Cxcl13* (M2 macrophage marker). n = 4 (four mice for each type of hydrogel), mean values ± s.d. Statistical analysis: one-way ANOVA with Tukey post-test, $^{\#}p < 0.05$ and $^{*}p < 0.01$.

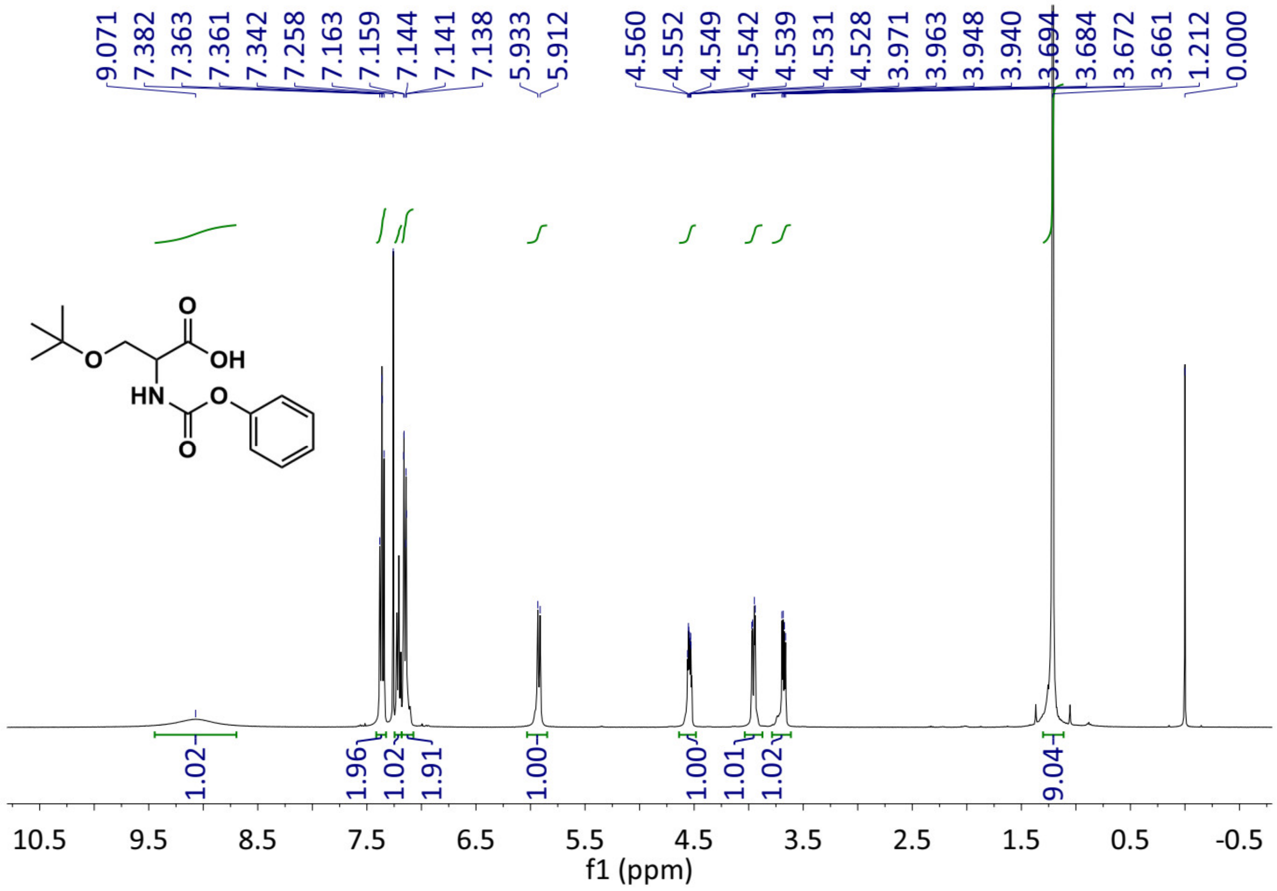

Supplementary Figure 8. ^{1}H NMR (400 MHz, $CDCl_3$) spectrum of 3-tert-butoxy-2(phenoxycarbonyl-amino)propionic acid.

Supplementary Tables

Supplementary Table 1. Characterization of PSer hydrogel precursor polymers.

	DP[a]	Mn (Da)	Đ[b]
PSerDA short chain	36	3300	1.12
PSerDA long chain	97	8600	1.18

[a] DP means the average degree of polymerization that was calculated from ^{1}H NMR characterization in D_2O. [b] Đ means the dispersity that was measured by GPC.

Supplementary Table 2. The components for hydrogel preparation.

Hydrogel	Components
PEG2000	20 wt% PEGDA2000 in H_2O, with 0.1 wt% photoinitiator 2-Hydroxy-4'-(2-hydroxyethoxy)-2-methylpropiophenone (I2959)
PEG5000	20 wt% PEGDA5000 in H_2O, with 0.1 wt% I2959
PEG5000-H	4 wt% PEGDA5000 in H_2O, with 0.1 wt% I2959
PSer3300	20 wt% PSer3300 in H_2O, with 0.1 wt% I2959
PSer8600	20 wt% PSer8600 in H_2O, with 0.1 wt% I2959
PAM1	19 wt% acrylamide and 1 wt% N,N'-Methylenebisacrylamide in DMSO, with 0.1 wt% I2959
PAM2	18 wt% acrylamide and 2 wt% N,N'-Methylenebisacrylamide in DMSO, with 0.1 wt% I2959

Supplementary Table 3. Elemental compositions of hydrogels determined from XPS.

Hydrogels	Elemental compositions (%)					
	C1*s*				O1*s*	N1*s*
	C-C	C-O/C-N	CO-O	Total		
PSer3300	21.42	23.43	14.61	59.46	12.76	27.78
PSer8600	15.64	25.95	16.58	58.17	13.80	28.03
PEG2000	-	-	-	67.74	32.26	-
PEG5000	-	-	-	66.67	33.33	-

Supplementary Table 4. The mouse cytokine array coordinates.

Coordinate	Target/Control	Alternate Nomenclature		Coordinate	Target/Control	Alternate Nomenclature
A1, A2	Reference Spot	——		C17, C18	IL-16	——
A23, A24	Reference Spot	——		C19, C20	IL-17	——
B1, B2	BLC	CXCL13/BCA-1		C21, C22	IL-23	——
B3, B4	C5/C5a	Complement Component 5a		C23, C24	IL-27	——
B5, B6	G-CSF	——		D1, D2	IP-10	CXCL10/CRG-2
B7, B8	GM-CSF	——		D3, D4	I-TAC	CXCL11
B9, B10	I-309	CCL1/TCA-3		D5, D6	KC	CXCL1
B11, B12	Eotaxin	CCL11		D7, D8	M-CSF	——
B13, B14	sICAM-1	CD54		D9, D10	JE	CCL2/MCP-1
B15, B16	IFN-γ	——		D11, D12	MCP-5	CCL12
B17, B18	IL-1α	IL-1F1		D13, D14	MIG	CXCL9
B19, B20	IL-1β	IL-1F2		D15, D16	MIP-1α	CCL3
B21, B22	IL-1ra	IL-1F3		D17, D18	MIP-1β	CCL4
B23, B24	IL-2	——		D19, D20	MIP-2	CXCL2
C1, C2	IL-3	——		D21, D22	RANTES	CCL5
C3, C4	IL-4	——		D23, D24	SDF-1	CXCL12
C5, C6	IL-5	——		E1, E2	TARC	CCL17
C7, C8	IL-6	——		E3, E4	TIMP-1	——
C9, C10	IL-7	——		E5, E6	TNF-α	——
C11, C12	IL-10	——		E7, E8	TREM-1	——
C13, C14	IL-13	——		F1, F2	Reference Spot	——
C15, C16	IL-12 p70	——		F23, F24	PBS (Negative Control)	Control (-)

Supplementary Table 5. Mouse specific forward and reverse primer sets used for qPCR analysis of RNA levels.

Gene	Primers (5' to 3'): Sense & Antisense
Col1a1	Forward: 5'-CATGTTCAGCTTTGTGGACCT-3'
	Reverse: 5'-GCAGCTGACTTCAGGGATGT-3'
CD11b	Forward: 5'-CCAAGAGAATGCAAAAGGCTTT-3'
	Reverse: 5'-GGGGGGCTGCAACAACCACA-3'
CD68	Forward: 5'-GCCCGAGTACAGTCTACCTGG-3'
	Reverse: 5'-AGAGATGAATTCTGCGCCAT-3'
Ly6g	Forward: 5'-TGCCCCTTCTCTGATGGATT-3'
	Reverse: 5'-TGCTCTTGACTTTGCTTCTGTGA-3'
Ccl19	Forward: 5'-GGGGTGCTAATGATGCGGAA-3'
	Reverse: 5'- CCTTAGTGTGGTGAACACAACA-3'
Cxcl13	Forward: 5'-GGCCACGGTATTCTGGAAGC -3'
	Reverse: 5'-GGGCGTAACTTGAATCCGATCTA-3'

1 Yang, Z., Mao, Z. & Ling, J. Phosgene-free synthesis of non-ionic hydrophilic polyserine. *Polym. Chem.* **7**, 519-522, (2016).
