# Supplementary material for: Bio-inspired poly-DL-serine materials resist the foreign-body response
Source: Nat Commun. 2021 Sep 7;12:5327. doi: 10.1038/s41467-021-25581-9 (PMC8423817; doi:10.1038/s41467-021-25581-9)
Supplement: Supplementary file 3 — Reporting Summary [file 41467_2021_25581_MOESM3_ESM.pdf]

nature portfolio

Corresponding author(s): Runhui Liu

Last updated by author(s): Jul 7, 2021

Reporting Summary

Nature Portfolio wishes to improve the reproducibility of the work that we publish. This form provides structure for consistency and transparency in reporting. For further information on Nature Portfolio policies, see our Editorial Policies and the Editorial Policy Checklist.

Statistics

For all statistical analyses, confirm that the following items are present in the figure legend, table legend, main text, or Methods section.

n/a	Confirmed	
☐	☒	The exact sample size (*n*) for each experimental group/condition, given as a discrete number and unit of measurement
☐	☒	A statement on whether measurements were taken from distinct samples or whether the same sample was measured repeatedly
☐	☒	The statistical test(s) used AND whether they are one- or two-sided<br>*Only common tests should be described solely by name; describe more complex techniques in the Methods section.*
☒	☐	A description of all covariates tested
☐	☒	A description of any assumptions or corrections, such as tests of normality and adjustment for multiple comparisons
☐	☒	A full description of the statistical parameters including central tendency (e.g. means) or other basic estimates (e.g. regression coefficient) AND variation (e.g. standard deviation) or associated estimates of uncertainty (e.g. confidence intervals)
☐	☒	For null hypothesis testing, the test statistic (e.g. *F*, *t*, *r*) with confidence intervals, effect sizes, degrees of freedom and *P* value noted<br>*Give P values as exact values whenever suitable.*
☒	☐	For Bayesian analysis, information on the choice of priors and Markov chain Monte Carlo settings
☐	☒	For hierarchical and complex designs, identification of the appropriate level for tests and full reporting of outcomes
☒	☐	Estimates of effect sizes (e.g. Cohen's *d*, Pearson's *r*), indicating how they were calculated

Our web collection on statistics for biologists contains articles on many of the points above.

Software and code

Policy information about availability of computer code

Data collection	AVANCE III 400 spectrometer (400 MHz) was operated with TopSpin software (version: 3.1);<br>Waters XEVO G2 TOF mass spectrometer with MassLynxTM software (version: 4.1);<br>Waters GPC instrument was operated with Breeze 2 software (version: 6.20.00.00);<br>Chirascan CD spectrometer was operated with Pro-Data Viewer software (version: 4.2.6);<br>Thermo Scientific K-Alpha XPS System with Avantage software (version: 5.948);<br>Hitachi Limited S-4800 Field Emision SEM operated with FE-PC SEM software (version: 3.18);<br>Universal testing machine (AI-3000, Gotech Testing Machines Co., Ltd.) was operated with U62 software (version: 6.1.1.59);<br>Pannoramic 250/MIDI equipped with the CaseViewer software (version: 2.0);<br>ImageQuant LAS 4000 (version: 1.2);<br>BGISEQ-500 platforms;<br>Primers were designed and evaluated using NCBI Primer-BLAST tool;<br>CFX96 Real-Time System was operated with Bio-Rad CFX Manager software (version: 2.1.1022.0523).

Data analysis	Excel (version: 16.0.11727.20222);<br>Origin (version: 8.5.0 SR1 b161);<br>MestReNova (version: 14.0.0-23239);<br>XPS Peak Fit (version: 4.1);<br>CaseViewer (version: 2.0);<br>Adobe Photoshop CC (version: 20.0.6)<br>ImageQuant LAS 4000 Control software (version: 8.1).

For manuscripts utilizing custom algorithms or software that are central to the research but not yet described in published literature, software must be made available to editors and reviewers. We strongly encourage code deposition in a community repository (e.g. GitHub). See the Nature Portfolio guidelines for submitting code & software for further information.

Data

Policy information about availability of data

All manuscripts must include a data availability statement. This statement should provide the following information, where applicable:

- Accession codes, unique identifiers, or web links for publicly available datasets
- A description of any restrictions on data availability
- For clinical datasets or third party data, please ensure that the statement adheres to our policy

Data that support the findings detailed in this study are available in the Supplementary Information and this article. RNA-seq data have been deposited in the NCBI Gene Expression Omnibus database under accession code PRJNA699958 [https://www.ncbi.nlm.nih.gov/sra?linkname=bioproject_sra_all&from_uid=699958]. The Source data underlying Figures. 4b, c, d, f, g, h, 5b, d, 6a, b, c, d, e, g, Supplementary Figures. 3b, 4, 5c, and 7 are provided as a Source data file. Any other source data perceived as pertinent are available, on reasonable request, from the corresponding author.

Field-specific reporting

Please select the one below that is the best fit for your research. If you are not sure, read the appropriate sections before making your selection.

☒ Life sciences ☐ Behavioural & social sciences ☐ Ecological, evolutionary & environmental sciences

For a reference copy of the document with all sections, see nature.com/documents/nr-reporting-summary-flat.pdf

Life sciences study design

All studies must disclose on these points even when the disclosure is negative.

Sample size	No sample size calculation was performed in advance. Samples size are determined according to comparable experiments in previously articles.
Data exclusions	No data were excluded from the analyses in this study.
Replication	In vivo experiments were performed once. For in vivo experiment, reproducibility was verified by similar results for individual animal within the group. IACUC approval requires that the minimum possible number of animals be used that yield statistically significant results.
Randomization	Randomization used for in vivo study. For experiments other than those performed in vivo, such as compressive test and water content test, randomized conditions were not necessary.
Blinding	All in vivo experiments were blinded for data processing. For experiments other than those performed in vivo, experiments were not blinded.

Reporting for specific materials, systems and methods

We require information from authors about some types of materials, experimental systems and methods used in many studies. Here, indicate whether each material, system or method listed is relevant to your study. If you are not sure if a list item applies to your research, read the appropriate section before selecting a response.

Materials & experimental systems

n/a	Involved in the study	
☐	☒	Antibodies
☒	☐	Eukaryotic cell lines
☒	☐	Palaeontology and archaeology
☐	☒	Animals and other organisms
☒	☐	Human research participants
☒	☐	Clinical data
☒	☐	Dual use research of concern

Methods

n/a	Involved in the study	
☒	☐	ChIP-seq
☒	☐	Flow cytometry
☒	☐	MRI-based neuroimaging

Antibodies

Antibodies used	rabbit anti-mouse F4/80 monoclonal antibody (Cell Signaling Technology; catalog no. 30325);<br>Alexa Fluor® 488-conjugated goat anti-rabbit IgG (H+L) antibody (Servicebio; catalog no. GB25303);<br>mouse anti-mouse αSMA monoclonal antibody (Servicebio; catalog no. GB13044);<br>Alexa Fluor® 488-conjugated goat anti-mouse IgG (H+L) antibody (Servicebio; catalog no. GB25301);<br>rabbit anti-mouse CCR7 monoclonal antibody (Novus Biologicals; catalog no. NBP2-67324);<br>rabbit anti-mouse TNF-α polyclonal antibody (Servicebio; catalog no. GB11188);<br>goat anti-mouse IL-12 polyclonal antibody (Novus Biologicals; catalog no. NB600-1443);<br>goat anti-mouse IL-10 polyclonal antibody (Novus Biologicals; catalog no. AF519);<br>HRP-labeled goat anti-rabbit antibody (Servicebio; catalog no. GB23303);<br>HRP-labeled rabbit anti-goat antibody (Servicebio; catalog no. GB23204).
Validation	For each antibody used in this study, validations and applications are stated on the manufacturer's websites:<br>rabbit anti-mouse F4/80 monoclonal antibody: Species Reactivity: Mouse; Application: WB, IP, IF;<br>Alexa Fluor® 488-conjugated goat anti-rabbit IgG (H+L) antibody: Application: IF;<br>mouse anti-mouse αSMA monoclonal antibody: Species Reactivity: Human, Mouse, Rat, Rabbit; Application: WB, IHC, IF;<br>Alexa Fluor® 488-conjugated goat anti-mouse IgG (H+L) antibody: Application: IF;<br>rabbit anti-mouse CCR7 monoclonal antibody: Species Reactivity: Human, Mouse, Rat; Application: WB, ICC/IF, IHC, IHC-P, IP, KD;<br>rabbit anti-mouse TNF-α polyclonal antibody: Species Reactivity: Mouse; Application: WB, IHC, IF;<br>goat anti-mouse IL-12 polyclonal antibody: Species Reactivity: Mouse; Application:WB, ELISA, ICC/IF, IHC, IHC-P;<br>goat anti-mouse IL-10 polyclonal antibody: Species Reactivity: Mouse, Rat; Application: WB, B/N, ICC/IF;<br>HRP-labeled goat anti-rabbit antibody: Application: IHC;<br>HRP-labeled rabbit anti-goat antibody: Application: IHC.

Animals and other organisms

Policy information about studies involving animals; ARRIVE guidelines recommended for reporting animal research

Laboratory animals	Male mice (C57/BL6 6 weeks old) used in this study. Mice were raised in an IVC system at 20-26 °C and 40-70% humidity, with a dark/light cycle of 12 h.
Wild animals	The study did not involve wild animals.
Field-collected samples	The study did not involve samples collected from the field.
Ethics oversight	All animal procedures were performed in accordance with the Guidelines for Care and Use of Laboratory Animals of the Ninth People's Hospital, and experiments were approved by the Animal Ethics Committee of the Ninth People's Hospital, Shanghai Jiao Tong University School of Medicine.

Note that full information on the approval of the study protocol must also be provided in the manuscript.
